# Potential of Liquid Extraction Surface Analysis Mass Spectrometry (LESA—MS) for the Characterization of Polymer-Based Materials

**DOI:** 10.3390/polym11050802

**Published:** 2019-05-05

**Authors:** Ambre Issart, Joanna Szpunar

**Affiliations:** Institute of Analytical and Physical Chemistry for the Environment and Materials (IPREM), UMR 5254CNRS—UPPA, Hélioparc, 2, av. Pr. Angot, 64053 Pau, France; ambre.issart@univ-pau.fr

**Keywords:** liquid extraction surface analysis, nanoelectrospray, polymers, mass spectrometry

## Abstract

Liquid extraction surface analysis mass spectrometry (LESA -MS) is a direct analysis method suitable for the analysis of polymers. It is based on a fast and efficient extraction of polymer components, such as non-intentionally added species (NIAS), post-polymerization residues, or additives, and residues resulting from specific uses followed by their MS detection. In comparison with batch methods, it is a “green” method, using negligible volumes of organic solvents, and it is cost-effective, avoiding lengthy sample preparation procedures. It can be used for the detection of known molecules (targeted analysis), identification of unknown species (exploratory analysis requiring MS/MS) and semi-quantative analysis, if standards are available. The to-date applications of LESA-MS in the field of polymer science are reviewed and critically discussed taking into account the hands-on experience from the authors’ laboratory. Future possibilities of LESA applications are highlighted.

## 1. Introduction

Polymers are mainly petroleum-based and usually contain additives that ensure their mechanical properties and attractive appearance are retained, but also improve their life-time and lower processing costs [1,2,3,4]. Polymers are omnipresent in everyday life, used in clothing to electronics, and in medical equipment to food packaging. Therefore, concerns about polymer-related risks are increasingly studied [5,6,7]. The development of new polymer-based materials requires their characterization and safety control. Safety aspects are crucial for polymers and plastics used in medical applications, food contact materials, children’s toys, and waste. Moreover, with the increase in the use of recycled materials, contamination can reach almost any domain [8,9]. Particular attention is focused on additives as they can easily diffuse throughout the polymer and are in direct contact with consumers [10,11]. Examples of polymer additives that have to be characterized include lubricants, surfactants, antistatic agents, antioxidants, plasticizers, toughening agents, and adhesion promoters [12,13,14]. Other chemical species, which are added non-intentionally, can represent hazards. Polymers and additives are sensitive to high temperature and thus degrade when they are processed, producing smaller molecules and creating residues, pollution and odor. Some of these pollutants can be post-polymerization residues (by-products, catalysts, monomers, etc.) or come from polymer recycling or reactions with chemicals (e.g., dishwashing). Possible contamination of recycled materials needs to be scrutinized [8,9]. Moreover, even if some additives are not harmful in their initial state, their degraded version or metabolites can be.

The various origins of contaminants make plastic final composition hard to predict. As a consequence, polymeric materials should be carefully analyzed to assess the presence of species able to show any toxic effects. Considering the increasing complexity of synthetic polymer-based materials and the huge diversity of additives used in polymer formulations, it is necessary to develop methods to characterize those complex materials and, more specifically, their surface. The contaminants usually have lower viscosities than the polymer they are stabilizing and they are located at the surface of the material (viscosity segregation). Also, another type of segregation within the polymer matrix is based on architectural differences. For example, in the case of two polymers of the same chemical unit but with different chain architecture (linear versus cyclic), surface enrichment may occur [15]. Moreover, surface contamination can modify surface properties. For example, the migration of lubricants to the surface will change its adhesion, whereas that of light-sensitive components can cause color changes [16]. As a consequence, surface analysis of polymeric materials can provide information about the chemical structure of the polymer, the additives, and also any possible contaminants.

Mass spectrometry is increasingly used as an efficient tool for the characterization of synthetic polymers, in terms of molecular weight, monomer sequence, terminal groups, additives, etc. [17,18,19]. The progress in soft ionization techniques, such as electrospray ionization (ESI) [20], matrix-assisted laser desorption/ionization (MALDI), desorption electrospray ionization (DESI) and direct analysis in real time (DART) [21,22,23], and large molecular mass range detection (time-of-flight MS) opened new possibilities to polymer analysis, offering not only complementary information to conventional methods but also new possibilities (e.g., surface mapping, on-site analysis, etc.) [21,22,23]. Indeed, many polymers are hard to solubilize. For example, some polyolefin (isotactic PP, HDPE and LDPE) are insoluble at room temperature (without degrading the polymer), making their analysis very complicated with conventional methods. Hence, their direct analysis and surface chemical mapping offer a promising solution. A common shortcoming of these methods is sample preparation. The extraction method for polymer and polymer additives is often a critical parameter. Extraction followed by chromatographic separation allows the reduction of sample complexity. However, it can considerably increase analysis time and is not adapted to every type of material as the solubility of the sample to be analyzed is a critical factor. On the other hand, MALDI-MS requires the deposition of a matrix over the sample to facilitate desorption and ionization. In this case, photo-dissociation can increase in-source fragmentation, complicating spectral analysis [24].

DESI-MS and DART-MS desorb analyte molecules from samples by ionizing their surfaces with charged aerosol or ionizing gas, respectively. The sample is analyzed without pre-treatment. DESI uses an electrically charged aerosol, directed toward the object, which is created by pneumatically-assisted electrospray of a solvent containing an electrolyte at low concentration. The highly charged aerosol droplets hit the surface to be analyzed and thus cover it with a liquid film [25]. DART uses a heated gas stream carrying ions formed in a plasma discharge [21]. Both methods are complementary as one can deal with ionic species (DESI) and the other one can ionize non-polar neutral ones (DART). Both DESI and DART require high temperatures and may lead to polymer degradation; degradation products can thus be detected, falsifying the analysis of the material’s intrinsic contaminants.

Liquid extraction surface analysis mass spectrometry (LESA-MS) is a relatively new ambient direct analysis method, developed by the Van Berkel group [26]. Similarly to other ambient MS techniques, it can be performed under atmospheric conditions and no sample pre-treatment is needed (other than cutting the (solid) sample). The technique separates the desorption and ionization steps. The extraction of the analytes requires only a small volume of solvent unlike MALDI or DESI-MS which require an addition of the matrix or a sample swelling before the analysis, respectively. Even though DESI-MS does not require any sample preparation, its process of analyte extraction involves the creation of charged microdroplets which wet and swell the sample surface, creating a solid–liquid interface (the analytes are extracted at this interface).

The method has a wide range of applications in metabolite, lipid, protein, and carbohydrate analysis [27,28,29,30,31], and it is starting to be applied in polymer sciences [32,33,34,35,36]. The objective of this review is to critically discuss the current LESA-MS applications for polymer analysis, its advantages and limitations, and to highlight the possibilities of widening its role in this field.

## 2. LESA—Operation Principle and Critical Parameters

**Figure 1:**
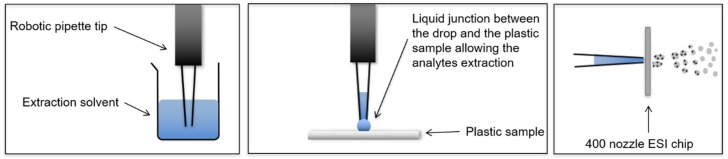
Liquid extraction surface analysis mass spectrometry LESA-MS principle.

The LESA-MS system is commercially available from Advion (Ithaca, NY, USA). It works using a robotic system that allows a nanoelectrospray infusion of samples dissolved in an aqueous organic solvent (Figure 1). The sample is placed on a plate and any part of its surface can be analyzed by a two-step procedure involving (i) the extraction of analytes into a hanging solvent drop by the creation of a micro-junction between the solvent drop and the sample and (ii) spraying the solvent (containing the extracted analytes) through a nanoESI silicon-based chip. The use of a nano-ESI chip allows the creation of a long-lasting spray from very low amounts of sample (at a rate of approximately 100–200 nL min-1 (3 µL of sample can be sprayed for 15 min).

The optimization of LESA-MS for polymer analysis involves several parameters: (i) the composition of the solvent and its volume (aspired and dispensed), (ii) the tip height above the sample, (iii) the contact time between the solvent and the surface, (iv) the aspiration speed, (v) the number of repetitions of the extraction operation and, finally, (vi) the pressure and voltage applied during the spray. As a consequence, many different combinations of parameters have to be tested in order to achieve the most efficient extraction. The LESA-MS parameters used in polymer-related studies are summarized in Table 1.

The main limitation of the method is the necessity to create a micro-junction: it can be difficult on rough, wettable or absorbent surfaces. It works best on homogeneous hydrophobic surfaces, making the method highly compatible for many synthetic polymers [37]. The solvent composition was optimized for the analysis of polymer films used for food packaging to achieve a stable liquid micro-junction between the solvent droplet and the polyester surface, ensuring sufficient swelling of the polymer substrate to extract the analytes and generate a stable ion current from the electrospray source [36]. The optimum solvent mixtures contained MeOH providing a stable electrospray, chloroform responsible for swelling of the polymer and facilitating extraction and formic acid (for positive-ion mode ESI-MS) aiding protonation of the analytes (increasing the abundance of ions for detection) [36]. In the case of polymers, analyte desorption involves simultaneous swelling and extraction. The possibility to change the solvent allows targeting the extraction of a chosen component rather than another.

The dispension and aspiration height depends on the hardness, wettability and composition of the surface. Optimizing these parameters allows keeping a great micro-junction (i.e., a good extraction).

The number of repetitions and the time of the extraction should also be optimized knowing that there are some composition variations (due to the use of a solvent drop): a compromise between acceptable extraction and a stable micro-junction has to be found [36]. The spray voltage and the pressure also have to be adapted to the method but most of the time it relies on the supplier guidelines for individual solvents.

Other more specific parameters can be modified such as the dispension or aspiration speed or the possibility to aspirate air after the sample (preventing loss of the drop from the tip between the sample and the mass spectrometer).

An important point to be considered while carrying out LESA analysis is the sample homogeneity. It is considered to be a critical parameter as the size of the drop is tiny in comparison with the size of most plastic samples. Hence, the studied sample has to be homogeneous to consider the small extraction area as representative for the whole material surface. Alternatively, the method can be used to detect sample inhomogeneity and characterize specific points of, e.g., polymer devices.

## 3. Application Areas

### 3.1. Characterisations of Polymer Matrices

LESA-MS is a powerful tool in the quality control of polymers able to detect post-polymerization residues and products of polymer degradation. Oligomers migrating from low-quality low-density polyethylene (LDPE) candidate food film packaging were detected [36]. The presence of easily migrating products of polymer degradation (e.g., resulting from UV exposure) or created during high temperature processing (e.g., manufacturing or recycling) was also demonstrated [38]. The release of small molecules able to migrate and be released from the polymer surface as a result of any process that the polymer can undergo in its lifetime can be easily detected by this technique.

**Figure 2:**
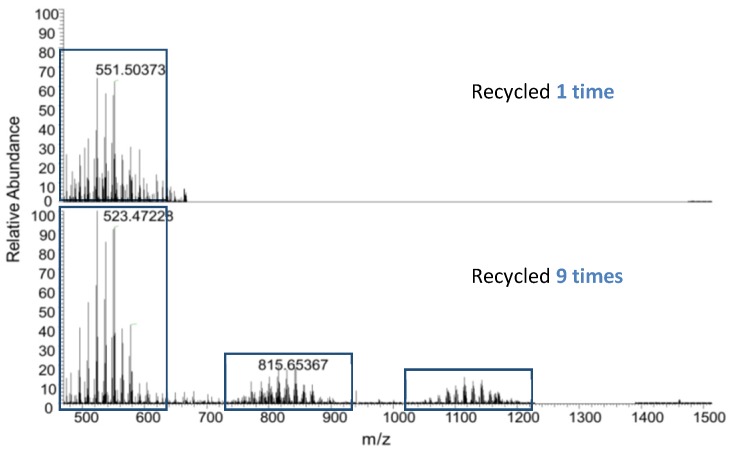
LESA-MS analysis on recycled bare polyethylene: upper panel - one cycle, bottom panel - nine cycles. (Reprinted from [36] with the permission of Elsevier.)

The LESA-MS method was applied to the direct analysis of polymers intended to be used as food packaging: polypropylene (PP) and low-density olyethylene (LDPE) [36]. These materials are among the most often used for packaging in contact with food. The study focused on the possible migration of polymeric residues; the studied sample was free of any additives and thus unstable. The analysis revealed the formation of low molecular species during recycling (realized by consecutive extrusion). Their presence correlated with the number of recycling cycles; example spectra are shown in Figure 2.

The peak clusters in Figure 2 are composed of peaks with a m/z difference of 14 Da, which corresponds to the LDPE (or PP) monomer. Very similar results were obtained when analyzing the polypropylene matrix [36]. Polyolefin thermal degradation is known to engender mainly volatile toxic compounds [39,40]. However, the LESA-MS analyses of non-stabilized polyolefin surfaces showed that they also generated non-volatile molecules: smaller pieces of polymers. These pieces of polymers correspond to n-alkanes/alkenes up to 1200 Da. Indeed, when in contact with oxygen and elevated temperatures, polyolefin chains tend to break to form those oligomers [41].

**Figure 3:**
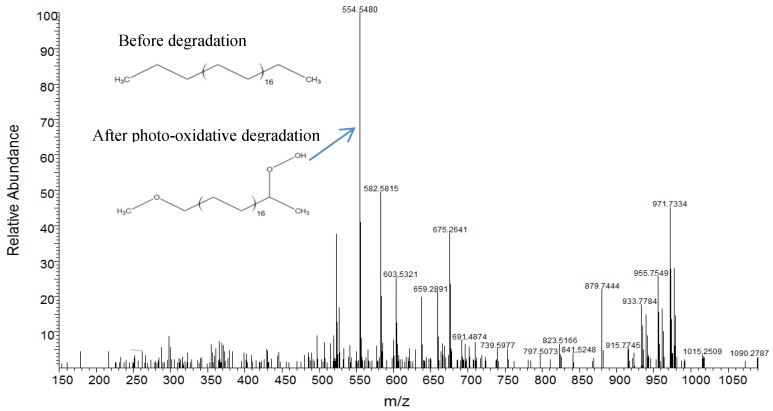
LESA-MS spectrum of an oxidized low-quality low-density polyethylene (LDPE). (Reprinted from [36] with permission of Elsevier).

Beside thermo-oxidative degradations, polyolefins also suffer from exposure to UV (photo-oxidative degradation). After exposure to UV light, the creation of a carboxylic group is very common. The potential of LESA-MS to evaluate polyolefin degradation during UV exposure was discussed. Additive-free LDPE was exposed to light at room temperature for 1 month and analyzed by LESA-MS (Figure 3) [36].

The mass spectrum revealed the presence of different degradation products such as alcohols or esters but the presence of a carboxylic group on the polymer chain was the most systematic. This stage of degradation is the one following oligomer formation described above (Figure 2). The oligomers can undergo further degradations and humidity, which is the case here. Then, the regularity of the oligomer chain observed in Figure 2 is lost due to chemical reactions, giving rise to new species (alcohols, ester, carboxylic acid, etc.). For example, the major peak from Figure 3 (m/z 554.5506 Da) is an oxidized molecule with a carboxylic function. Oxidized species are known to be more toxic than their non-oxidized equivalents.

**Figure 4:**
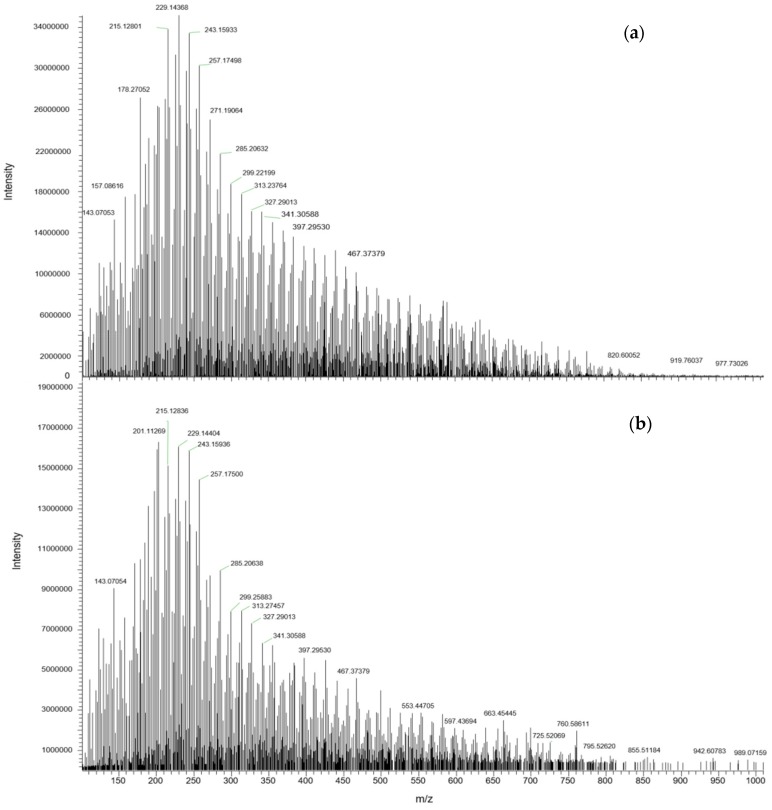
Direct infusion nano ESI-MS (**a**) vs. LESA-MS (**b**) of PE wax.

The information provided by LESA-MS is the same as that obtained by using traditional batch methods with solvent extraction (and usually subsequent evaporation preconcentration). Oxidized polyethylene wax was analyzed in infusion mode and compared with direct analysis by LESA-MS. The sample, synthesized by Radecka et al. [42], as a carbon source for the bacterial synthesis of polyhydroxyalkanoates (PHA), was solubilized in 0.1% formic acid in MeOH/CHCl_3_ (2:1 v/v) and infused directly (Figure 4a). The same solvent was used for LESA-MS (Figure 4b).

Figure 4 reveals that the same pattern was obtained by LESA-MS and by batch method (the spectrum obtained by LESA-MS is clearer). The series of peaks [M + H^+^] with a m/z difference of 14 Da is the same for both methods and the adducts [M + Na^+^] and [M + K^+^] were also identified in both spectra.

### 3.2. Detection of Polymer Additives

Additives are commonly used to improve the polymer’s mechanical properties and stability, and to lower manufacturing costs. They are usually small molecules and, as such, are able to diffuse within the polymer structure and off the polymer surface. As a result, they become common contaminants of food in contact with polymer packaging films or containers. The characterization of those components is a major issue in polymer quality assessment and control.

One of the biggest families of additives used for polymer stabilization is hindered amine light stabilizers (HALS). They are known to maintain aesthetic properties and stability during the polymer’s life [43]. HALS act as chain-breaking antioxidants, in which the initial molecule acts as a sacrificial component toward oxidation. The obtained molecule then acts as a free radical scavenger, allowing the turning of a free-radical into less harmful one. However, HALS suffer changes and loss of efficiency with time. HALS analysis was reported by MALDI-MS, DESI-MS [44], and electron spin resonance (ESR) [45], the latter unable to give information about the possible modification of the additive’s structure. LESA-MS was applied [35] to the monitoring of those additives in two separate studies: one following the chemical changes of two commercial HALS (TIN123 and TIN292) within cross-linked polyester or polyacrylate and the other one focused on the analysis of commercial HALS in polyester-based thermoset coil coating versus a coil coating with no stabilizer [34]. The study of the structural changes was of utmost importance as it is the key to understanding the HALS stabilization mechanisms; it could clearly benefit from using LESA-MS. The study realized on polyesters or polyacrylate allowed concluding that methylated HALS tends to deploy more easily in a non-pigmented coating: without pigments, UV-light penetrates easily in the matrix, allowing the “activation” of the methylated additive. In contrast, for highly pigmented coatings, HALS appeared to be almost inefficient [33].

**Figure 5:**
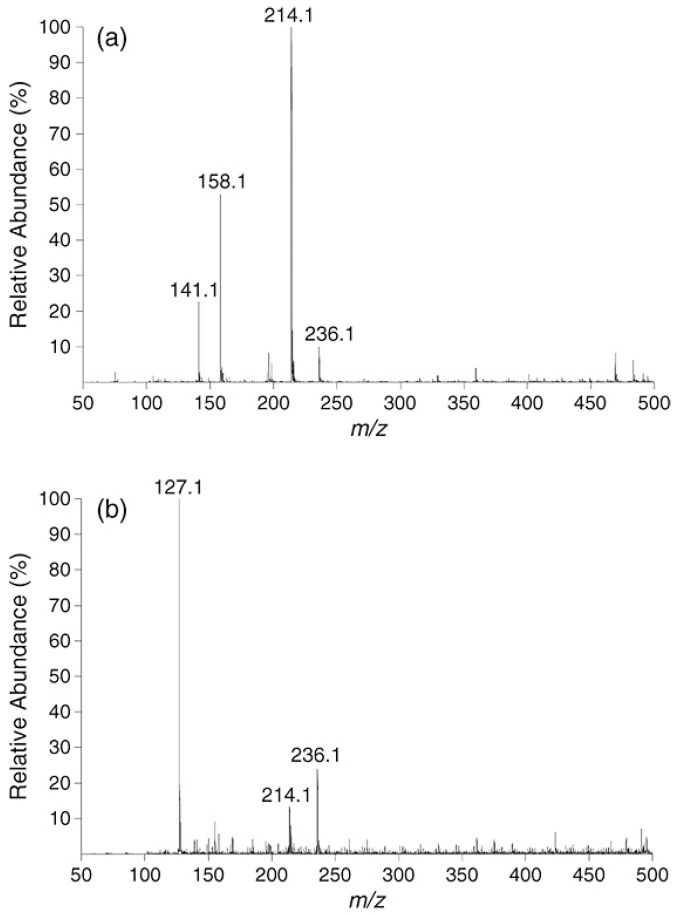
Positive-ion LESA-MS spectra obtained on pigmented polyester-based coil coatings formulated without HALS. (**a**) before outdoor exposure; (**b**) after 4 years of outdoor exposure. (Reprinted from [35] with permission of John Wiley and Sons.)

The other study assessed the degradation of HALS under harsh conditions (weathering conditions). Figure 5 compares the LESA-MS spectra of a sample (m/z 214.1 is a plasticizer) before (a) and after (b) 4 years of exposure to outdoors conditions (huge lowering of the amount of plasticizer and apparition of m/z 127.1 corresponding to a protonated melamine can be observed).

Also, LESA-MS was carried out on this weathered sample to try to relate the phenomenon of blooming with the HALS structural changes and linked it to the apparition of melamine (and its reaction with another component). Finally, two main degradation mechanisms of TIN123 could be proposed: N–O bond homolysis and cleavage of ester-linkage groups. Excellent reproducibility of LESA-MS method at difficult conditions (very low quantities, hard localization, etc.) was demonstrated; the analysis of seven different spots of the same panel showed an RSD of 5%. This study could take advantage of particular features of LESA-MS such as a possibility to carry out the analysis of low quantities hardly accessible material.

Another example of successful LESA-MS application is the analysis for brominated flame retardants (BFRs) [32]. In particular, tetrabromobisphenol A (TBBP-A) [46,47,48] was reported to accumulate in the body and was identified as an endocrine disrupter, being immunotoxic and carcinogenic [49,50,51]. Even though some of those BFRs are prohibited, they remain persistent due to plastic recycling and find their way to food packaging, toys, and medical materials. The usual screening methods for BFRs require long sample preparations (digestion, fractionation of the extracts, and separation by chromatography coupled to various detectors).

**Figure 6:**
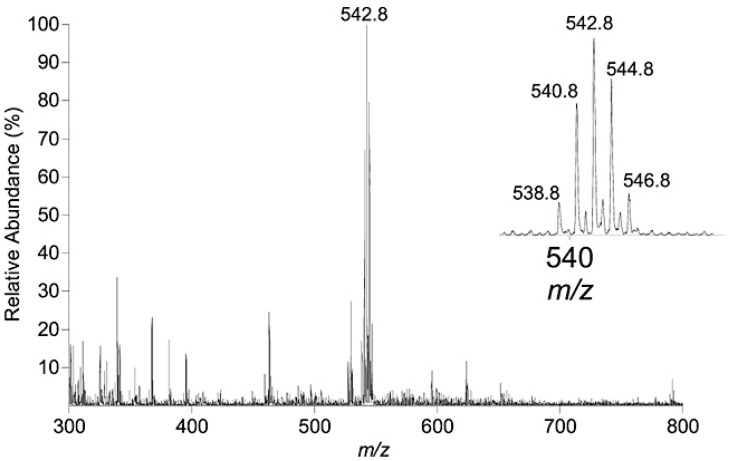
Negative-ion ESI-LESA-MS spectrum obtained for plastic e-waste. (Reprinted from [32] with permission of John Wiley and Sons.)

The study, published by Paine et al., by LESA-MS is the first one reporting the successful use of ESI-MS for the detection of BFRs. Nanoelectrospray ionization coupled to LESA-MS allowed simultaneous analysis for BFRs over a large range of polarities. A cluster of peaks corresponding to TBBP-A was observed during the analysis of a piece of e-waste intended to be recycled (Figure 6). Moreover, the e-waste pellets were divided according to their color and analyzed. The analysis was repeated eight times for each color, confirming the presence of TBBP-A in different amounts, and took only a few minutes to be realized thanks to the robotic system [32].

**Figure 7:**
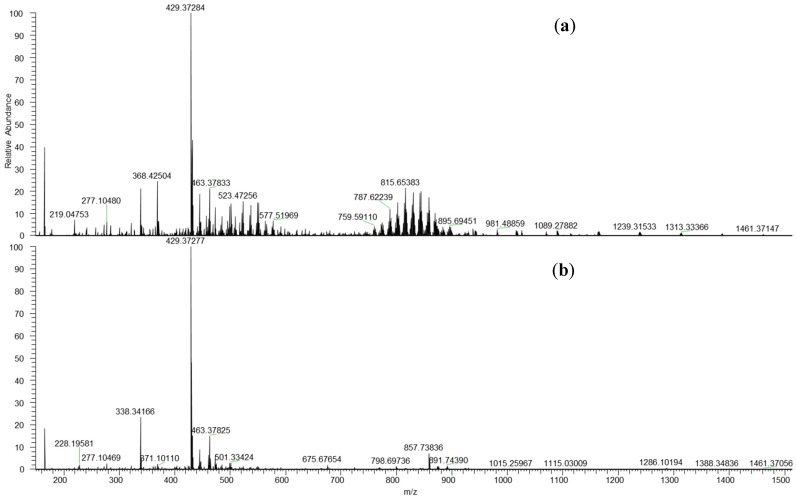
Spectra obtained after the analysis of two LDPE by LESA-MS (**a**) LDPE + 2.5% vitamin E; (**b**) LDPE + 2.5% vitamin E/C. (Reprinted from [36] with permission of Elsevier.)

LESA-MS was also applied to the detection of additives in “active” food packaging [36]. As the diffusion phenomena of additives through the polymer matrix are very hard to stop, synthetic additives (such as the previously described BFRs or BHT) with potential toxicity tend to be replaced by natural safer ones. Alpha-tocopherol (vitamin E), ascorbic acid (vitamin C) and condensed tannins were investigated as potential substituent for common synthetic additives [52,53]. They are delivered to the food thanks to the diffusion which is known as “active packaging” [54]. The migration of such a component may lead to a prolonged shelf life and preservation of the quality of the product [55,56,57,58,59]. The migration tests are very long and require specific conditions. LESA-MS was found to be a suitable technique for the comparison of polypropylene and LDPE stabilized with vitamin E/vitamin C and vitamin E/tannins and a non-stabilized one (Figure 7).

The LESA-MS screening of both blends revealed that the well-known synergistic effects between vitamin E and vitamin C provided a very good stabilization of the polymer: greatly lowering the migration of oligomers (created after degradation of polymers chains). No clusters of oligomers could be observed as in Figure 2 described earlier or as for LDPE compounded with vitamin E alone. Hence, in the formulation process, LESA-MS allowed choosing the best components for the stabilization of polymers to be found [36].

### 3.3. Species Resulting from Specific Use

**Figure 8:**
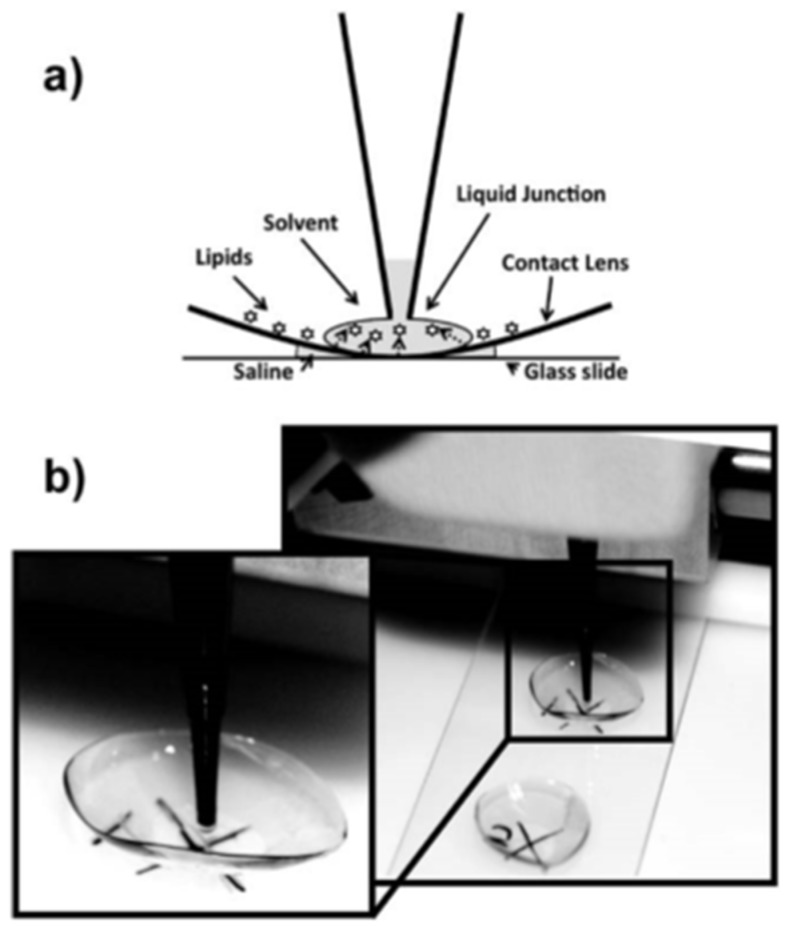
(**a**) Scheme of LESA extraction process on contact lenses; (**b**) Picture taken during extraction. (Reprinted from [33] with permission of the Royal Society of Chemistry of Public Analysts.)

LESA-MS was also applied to polymers used in other specific applications. An example is the application of LESA-MS for the medical field with the analysis of worn contact lenses and, more specifically, the extraction and determination of the biological deposits on the polymeric material [33]. The deposition of biological molecules on contact lenses can cause discomfort to the people wearing them. To overcome these problems, the nature of the deposited molecules has to be assessed as well as how the composition of the contact lens affects this deposition. So far, the analysis of lipid deposits on contact lenses was done through a bulk extraction of the soluble part of the lens. As a consequence, no discrimination could be done between the airside and the eyeside. LESA allows such discrimination. A lense was placed on a glass slide and analyzed by LESA-MS (Figure 8). The obtained results were compared to human tear and meibum samples used as standards and revealed that most of the lipids contained in the tears and the meibum were also found in both worn contact lenses.

The method showed many advantages for the identification of bio-residues on thin polymeric medical materials. Aside from being easy to set up and reproducible, LESA-MS of worn contact lenses allowed to achieve a non-destructive targeted analysis of lipid deposits thanks to the discrimination of both sides of the lens. This discrimination is not possible with traditional analysis methods as they require extraction processes from the whole lenses prior the analysis (processes that showed to degrade the polymer material, the analyte and thus increase the complexity and the chemical noise).

Another specific potential use of LESA-MS is the evaluation of food packaging after use and before being cleaned, recycled or reused; LESA-MS allows rapid assessment of the presence of fatty residues on the plastic surface [60].

## 4. Confirmatory (MS/MS) vs. Exploratory Aspects

The still unexplored potential of the LESA-MS methods is related to the possibility of its coupling to tandem mass spectrometry which offers the possibility of the formal identification of the studied species. For food packaging degradation studies, LESA-MS/MS allowed the confirmation of the chemical structure of the migrating alkanes (thermo-oxidation, Figure 2) and of the UV oxidized species (photo-oxidation, Figure 3). In the case of contact lenses, LESA-MS/MS gave access to profiles of the extracted species: mainly wax and cholesterol esters (CE) and polar lipids (phospholipids and fatty acids). Moreover, when LESA was coupled to a sensitive tandem mass spectrometer, extraction and mass spectrometric analysis could be achieved in a very short period of time, still producing accurate data. These data highlight the efficiency of LESA-MS/MS for the extraction and identification of bio-residues on polymeric medical materials with limited degradation of the material. The use of MS/MS on coil coatings samples was also reported [35]. As mentioned before, the analysis of non-stabilized coatings exposed to outdoor weathering revealed the presence of melamine. The use of LESA-MS/MS allowed the correlation of melamine presence with the blooming effect observed on degraded samples.

## 5. Semi-Quantitation

One of the advantages of LESA-MS is the possibility of semi-quantitative analysis. Brown et al. characterized 25 worn lenses and managed to identify, by LESA-MS/MS, 42 cholesterol esters with esterified acyl chains and demonstrated their variation in concentration without using a standard. The evaluation of the 20 most abundant cholesterol esters in two types of lenses was assessed and thanks to the cholesterol esters ions’ absolute abundances, concentration profiles were set up. It allowed them to conclude that, in spite of the similarities in the compositions of both lenses, the differences in abundances could show that lipid deposition on lenses depend on the lens itself.

**Figure 9:**
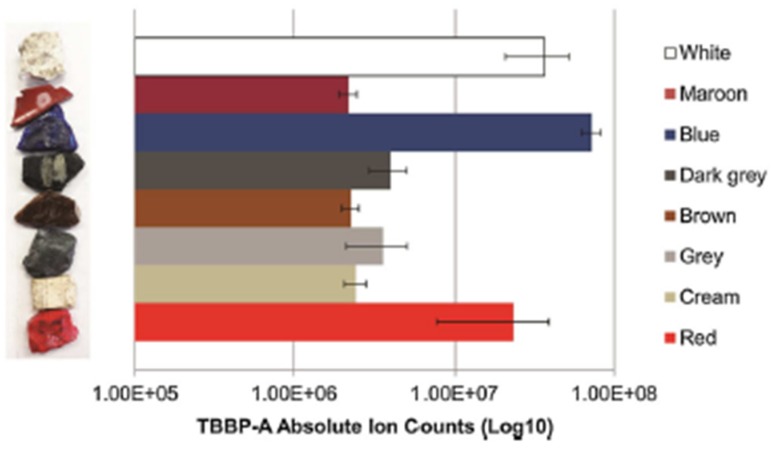
Histogram of the intensities obtained after the analyses of plastic e-wastes as a function of their color. The intensity of the ion m/z 542.8 corresponding to tetrabromobisphenol A (TBBP-A) was based on three replicate measurements. (Reprinted from [32] with permission of John Wiley and Sons.)

While studying e-wastes and the possible presence of BFR’s, Paine et al. demonstrated semi-quantitative reproducible analysis for TBBP-A by reporting TBBP-A absolute intensity (ion counts) according to the color of the plastic e-wastes (Figure 9).

Issart et al. obtained a LESA-MS calibration curve using a series of laboratory prepared solid state standards. Standards of low-density polyethylene were prepared by mixing different amounts of vitamin E in a concentration range corresponding to commercial samples (from 0.25 to 5%). The peak corresponding to vitamin E was quantified (m/z 429.373), revealing a linear evolution (R^2^ = 0.9948) of the intensity as a function of the amount of vitamin E [36]. Hence, migration assessments of vitamin E from the LDPE could be carried out and compared with an official method prescribed by EU regulations [2]. According to the official EU method, the food simulant (95% ethanol which simulates fatty food) must be analyzed by LC-MS after contact with the plastic sample. LDPE containing vitamin E was placed in a food simulant and analyzed. The study revealed a good correlation between the batch and LESA methods [60].

## 6. Conclusions

Liquid extraction surface analysis mass spectrometry (LESA-ESI-MS) is a direct analysis method suitable for the analysis of polymers. In comparison with batch methods, it is a “green” method, using negligible volumes of organic solvents, and is cost-effective, avoiding lengthy sample preparation procedures. It can be used for the detection of known molecules (targeted analysis), identification of unknown species (exploratory analysis requiring MS/MS) and semi-quantitative analysis. So far, LESA-MS and LESA-MS/MS have proven to be robust enough to clearly identify species from a broad range of complex polymer samples (e.g., contact lenses, dirty polymeric pellets, different types of food packaging, and coatings). These advantages open possibilities for future use where a fast evaluation of solid polymer samples is needed in the context of polymer quality control, aging and degradation, specific uses (medical, food-related, everyday consumer products) or recycling.

## Figures and Tables

**Table 1:** LESA-MS parameters used for polymer analyses.

Polymer Matrix	Analyte	Solvent Mixture	Contact Time (s)	Repetitions	Ref
Polyethylene, terephthalate (PET),	Polyethylene oxide (PEO)	1:1 H_2_O:ACN + 0.1% formic acid	8	-	[36]
polypropylene (PP) and polyethylene (PE)	Natural additives	2:1 MeOH:CHCl_3_ + 0.1% formic acid	8	-	[36]
Worn contact lenses	Lipids	(2:1 v/v) MeOH:CHCl_3_ + 8 mm CH_3_COONH_4_ (negative ion mode)	5	-	[33]
Worn contact lenses	Lipids	isoPrOH:MeOH:CHCl_3_ (4:2:1 v/v/v) + 20 mm CH_3_COONH_4_ (positive-ion mode)	5	-	[33]
Polyester-based coil coatings	HALS (additive)	MeOH:CHCl_3_ (2:1) + 0.1% formic acid (v/v)	1	3	[35]
Polyacrylate	HALS	MeOH:CHCl_3_ (2:1) + 0.1% formic acid (v/v)	1	2	[34]
Plastic e-wastes	Brominated flame-retardant (additives)	MeOH:CHCl_3_ (2:1) + CH_3_COONH_4_ (20 mm)	1	3	[32]

## Abreviations

LESA-nanoESI-MS	Liquid Extraction Surface Analysis nanoelectrospray Mass Spectrometry
NIAS	Non-Intentionally Added Species
ESI	Electrospray Ionization
MALDI	Matrix-Assisted Laser Desorption/Ionization
DESI	Desorption Electrospray Ionization
DART	Direct Analysis in Real Time
RSD	Relative Standard Deviation
PP	PolyPropylene
iPP	isotactic PolyPropylene
HDPE	High Density PolyEthylene
LDPE	Low Density PolyEthylene
PET	Polyethylene Terephthalate
PEO	PolyEthylene Oxyde
HALS	Hindered Amine Light Stabilizers
BFR	Brominated Flame-Retardant
TBBP-A	Tetrabromobisphenol A
CE	cholesterol esters
